# Involvement of the posterior limb of the internal capsule independently predicts the prognosis of patients with basal ganglia and thalamic hemorrhage

**DOI:** 10.3389/fneur.2024.1475444

**Published:** 2025-01-07

**Authors:** Sohan Gupta, Mengxuan Xiao, Na Liu, Yunxiao Zhao, Xiaolin Zhao, Yunqiang Huang, Yongming Wu, Zhenzhou Lin, Zhong Ji, Haihao Xu, Minzhen Zhu, Suyue Pan, Kaibin Huang

**Affiliations:** ^1^Department of Neurology, Nanfang Hospital, Southern Medical University, Guangzhou, China; ^2^Department of Neurology, Xuanwu Hospital, Capital Medical University, Beijing, China; ^3^Department of Neurology, Huadu District People's Hospital of Guangzhou, Guangzhou, China; ^4^Department of Neurology, Heyuan People's Hospital, Heyuan, China

**Keywords:** basal ganglia, thalamic, intracerebral hemorrhage, internal capsule, prognosis, max-ICH

## Abstract

**Background:**

Intracerebral hemorrhage (ICH) is the most lethal and devastating subtype of stroke. Basal ganglia hemorrhage and thalamic hemorrhage are the most common types of ICH, accounting for 50–70% of all ICH cases, leading to disability and death, and it involves the posterior limb of the internal capsule to varying degrees. In this study, we investigated the impact of varying degrees of the involvement of the posterior limb of the internal capsule on the prognosis of patients with basal ganglia and thalamic ICH and assessed whether it improves the predictive accuracy of the max-ICH score, an existing scale for ICH functional outcome.

**Methods:**

This is a multicenter, retrospective, observational study. We graded the involvement of the posterior limb of the internal capsule according to the degree of compression and injury (called iICH, ranging from 0 to 4). An unfavorable outcome was defined as a 90-day modified Rankin Scale (mRS) of > 2. Multivariate logistic regression analysis was used to identify independent risk factors associated with unfavorable prognosis. The discrimination was verified using receiver operating characteristic curve (ROC) analysis, while the calibration was verified by the Hosmer-Lemeshow test.

**Results:**

Of the 305 patients included, 188 from Nanfang Hospital were assigned to the development cohort, and 117 from Heyuan People's Hospital and Huadu District People's Hospital were assigned to the validation cohort. In the development cohort, iICH was identified as an independent factor of a 90-day unfavorable outcome, and the area under the ROC (AUC) was 0.774. When combined with the iICH, the AUC of max-ICH was significantly elevated from 0.816 to 0.866. Comparable results were found in the validation cohort.

**Conclusions:**

Increased involvement of the posterior limb of the internal capsule is associated with a worse outcome in patients with basal ganglia and thalamic ICH.

## 1 Introduction

Intracerebral hemorrhage (ICH) is the most lethal and devastating subtype of stroke (1, 2). Survivors have varying degrees of residual disability, and the functional independence rate is only 12–39% at follow-up (3), which represents a substantial financial burden on individuals and society. Basal ganglia hemorrhage and thalamic hemorrhage are the most common types of ICH, accounting for 50–70% of all ICH cases (4, 5). Due to the anatomically adjacent location, basal ganglia and thalamic ICH are prone to impair the internal capsule, especially the posterior limb of the internal capsule, which contains a large number of white matter fibers involved in the integration of motor and sensory inputs (6). Therefore, the impairment of the posterior limb of the internal capsule, either by direct pressure from the hematoma or secondary damage from hematotoxicity products (7), will lead to sequelae such as hemiplegia, hemianopsia, and sensory deficit (8–11). Clinical evidence has shown that motor outcome after stroke is closed related to the integrity of the corticospinal tract passing through the posterior limb of the internal capsule (6, 12–14), which further supports that the destruction of the posterior limb of the internal capsule by hematoma should be a key factor for unfavorable prognosis. Although current studies on the prognosis of ICH are not uncommon, however, few studies have addressed the involvement of the posterior limb of the internal capsule, which may affect the prognosis of patients with ICH and improve the predictive validity of existing models.

Minimally invasive surgery (MIS) is a promising treatment for deep cerebral hemorrhage with the advantages of short operative time and the accessibility to the clot while reducing damage to the fiber tract (15–17). In the recent Early Minimally Invasive Removal of Intracerebral Hemorrhage (ENRICH) trial (18), MIS has been shown to improve the prognosis of patients with ICH. Theoretically, in patients with basal ganglia and thalamic ICH, if the hematoma is only pressing on the posterior limb of the internal capsule but not penetrating (destroying), timely removal of the hematoma through MIS should be beneficial to the recovery of motor function. Therefore, it is necessary to study the influence of the degree of impairment to the posterior limb of the internal capsule on the prognosis of patients treated with MIS.

In this study, we first explored the relationship between different degrees of involvement of the posterior limb of the internal capsule and the prognosis of patients with basal ganglia and thalamic ICH in a development cohort and evaluated whether adding this indicator to an existing scale, maximally treated ICH (max-ICH) score (19), could improve its predictive efficacy. Then, we verified the reliability of the above results using a validation cohort. Finally, we evaluated the influence of different degrees of involvement of the posterior limb of the internal capsule on neurological prognosis in patients with MIS and conservative treatment, respectively.

## 2 Materials and methods

### 2.1 Study design and ethics statement

This is a multicenter, retrospective, observational study. The study protocol was approved by the Medical Ethics Committee of Nanfang Hospital (NFEC-2024-092) and has obtained the local ethics committee approval of the other two participating institutions. Given its observational and retrospective characteristics, informed consent was waived by the review committee and all data were fully de-identified. All study procedures and protocols complied with the Declaration of Helsinki.

### 2.2 Patient selection

We retrospectively reviewed data of consecutive ICH patients admitted to Nanfang Hospital, an academic hospital affiliated with Southern Medical University, from January 2016 to June 2021. Meanwhile, ICH patients admitted to the other two teaching hospitals, Heyuan People's Hospital and Huadu District People's Hospital, between March 2016 and February 2019 were screened for eligibility.

Patients were included if they met the following criteria: with ICH in the basal ganglia or thalamic region confirmed by cranial computed tomography (CT). Patients were excluded if they met one of the following criteria: (a) under the age of 18 or above the age of 85; (b) had secondary causes of ICH including aneurysm, malformations, cavernous vascular disease, venous sinus thrombosis, cerebral infarction hemorrhagic transformation, and oral anticoagulants; (c) had concurrent involvement of the posterior circulatory system by the hematoma (brainstem and cerebellum) or other lobar hemorrhages; (d) premorbid modified Rankin Scale (mRS) ≥ 2 points; (e) with tumor, cirrhosis, renal failure, or other diseases that cause a life expectancy of fewer than 3 months; (f) with incomplete data or lost to follow-up.

Patients from Nanfang Hospital were categorized into the development cohort, while patients from Heyuan People's Hospital and Huadu District People's Hospital were categorized into the validation cohort.

### 2.3 Candidate variables

Data of patients were obtained from electronic medical records by two trained resident doctors using uniformly standardized forms, including demographics (age and sex), medical history (hypertension, diabetes mellitus, heart disease, history of stroke), clinical rating scale [National Institutes of Health Stroke Scale (NIHSS), Glasgow Coma Scale (GCS), Acute Physiology and Chronic Health Evaluation II (APACHE II) score, and max-ICH score], baseline ICH volume, intraventricular hemorrhage, hematoma irregular shape, minimally invasive surgery, lateral ventricle puncture, and pulmonary infection during hospitalization.

The max-ICH score (19), whose items include NIHSS, age, intraventricular hemorrhage, anticoagulation, and ICH volume (lobar and non-lobar), is excellent and widely validated in predicting the prognosis of patients with cerebral hemorrhage (20–22).

### 2.4 Treatment during hospitalization

All ICH patients were managed in the stroke unit or neurocritical care unit during the acute phase and received standardized medical therapy and nursing care (23). All patients underwent continuous blood pressure monitoring and efforts were used to target the systolic blood pressure between 120 and 140 mmHg. Any underlying coagulation disorder was corrected as soon as possible. Venous thromboembolism was prevented by compression stockings and intermittent pneumatic compression devices. Hyperosmolar agents (mannitol) were given when CT scans showed mass effect and swelling or clinical signs showed increased intracranial pressure. Blood glucose levels and body temperature were kept as close to normal as possible.

About one-fifth of patients were treated with stereotactic minimally invasive surgery at Nanfang Hospital, while the patients at the other two hospitals were all treated conservatively. There are no widely accepted criteria for the surgery, and the following criteria were used for selecting candidates with basal ganglia and thalamic ICH for MIS in Nanfang Hospital: (a) Patients with hematoma size in basal ganglia ≥30 ml, hematoma size in thalamus ≥10 ml, or with severe neurological dysfunction (muscle strength ≤ Grade 2 or disturbance of consciousness with GCS score of ≤ 13) despite the hematoma size did not meet the above criteria; (b) 24–72 h after the onset of ICH; (c) Informed consent of the patient or legal representative were obtained.

### 2.5 The iICH grading scale and radiographic data

**Figure 1:**
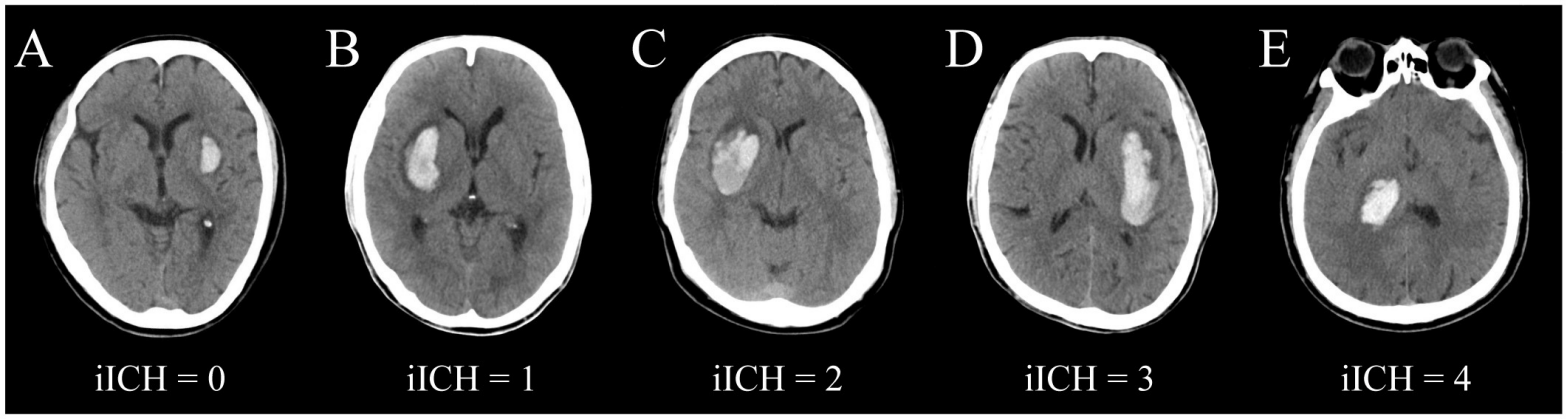
Schematic images of different grades of iICH. **(A)** iICH = 0, no involvement of the internal capsule; **(B)** iICH = 1, lateral ventricular wall width <1/2 contralateral lateral ventricular width; **(C)** iICH = 2, displacement of the lateral ventricular wall beyond the midline; **(D)** iICH = 3, hematoma damages the posterior limb of the internal capsule <1/3; **(E)** iICH = 4, hematoma damages the posterior limb of the internal capsule ≥1/3.

According to the degree of compression and penetrating injury to the posterior limb of the internal capsule by hematoma on cranial CT images, we constructed a scale called iICH. As the damage caused by a penetrating hematoma is likely to be more serious than that caused by a compressive hematoma, we defined penetrating injuries ≤ 1/3 and >1/3 posterior limb of the internal capsule as 3 and 4 points, respectively, complete compression injuries as 2 points, partial compression injuries as 1 point, and no involvement of the internal capsule as 0 point (Figure 1).

Two experienced neurologists, who were blinded to the participants' clinical characteristics and biochemical results, visually reviewed all CT images to calculate ICH volumes and assess iICH scores. Under the brain parenchymal window, they selected all the hematoma levels, carefully outlined the hematoma boundary manually according to the high density, and calculated the hematoma area layer by layer, then calculated the volume according to calculus methods. The regularity or irregularity of the hematoma was graded according to the scale established by Barras (24, 25). The concordance between the iICH scores rated by the two observers was good (kappa = 0.861, *p* < 0.001), and disagreements between them were resolved by joint discussion until a consensus was reached. Kendall's tau was used to assess the concordance of the ICH volumes, and its value was calculated to be 0.966 (*p* < 0.001), indicating good concordance. The means of the ICH volumes assessed by the two observers were ultimately used.

### 2.6 Primary outcome

Functional outcome at 90 days after ICH onset was the primary endpoint, where mRS of 3–6 points was considered as the unfavorable outcome and 0–2 as the favorable outcome. The mRS score at 3 months was obtained by two trained neurologists who were unaware of the other study data at the call-back interview.

### 2.7 Data analysis

Descriptive statistics were performed for patient characteristics. Categorical variables were expressed as the number of patients (n) and percentage (%), and compared using the two-sided Chi-square test or Fisher's test. Continuous data were presented as median [interquartile range (IQR)], and analyzed by Mann-Whitney *U*-test. Univariate analysis was conducted to screen factors associated with unfavorable prognosis, then candidate variables with a *p* < 0.05 were introduced into corresponding multivariate logistic regression models to identify independent risk factors associated with unfavorable prognosis, and the results were presented as odds ratios (ORs) with 95% confidence intervals (CIs). The predicted probability of the logistic regression model was used as a surrogate marker to construct the receiver operating characteristic (ROC) curve. The area under the ROC curve (AUC) was used as an accuracy index for evaluating the determination of independent risk factors in predicting 90-day unfavorable outcomes. The DeLong test was used to compare whether the AUCs between the models were significantly different. The fit of the model was evaluated by the Hosmer-Lemeshow test (26). All data were analyzed using SPSS 25.0 and GraphPad Prism 9.4.1. For all analyses, a two-sided *p*-value < 0.05 was considered to be statistically significant.

## 3 Results

### 3.1 Patient inclusion

**Figure 2:**
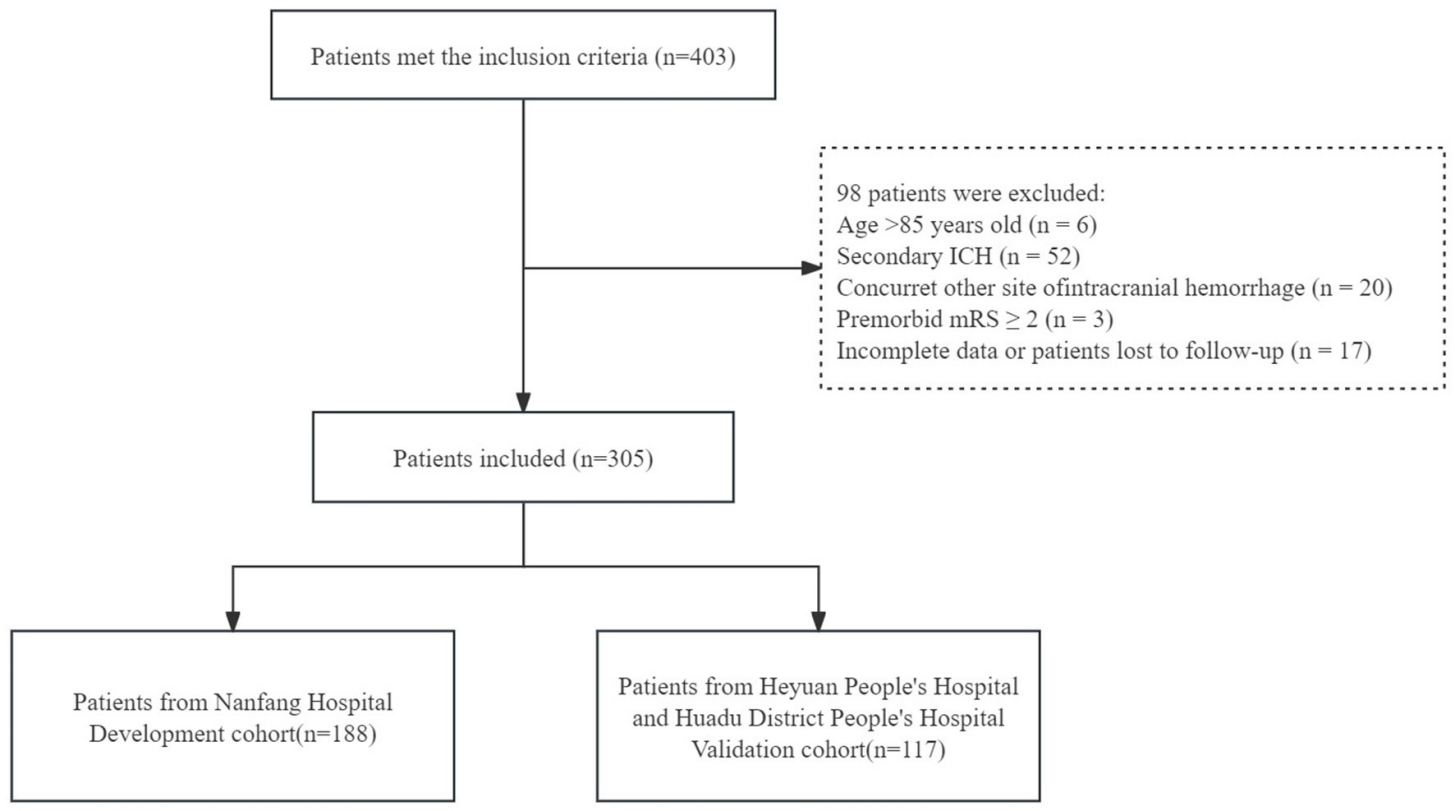
Patient inclusion flowchart.

After screening, a total of 305 patients from three centers were included in this study. The 188 patients from Nanfang Hospital were categorized into the development cohort, and the other 117 patients from Heyuan People's Hospital and Huadu District People's Hospital were categorized into the validation cohort (Figure 2).

### 3.2 The characteristics of development cohort

Table 1 shows the baseline characteristics of the patients in the development cohort. During follow-up, 108 (57.4%) patients had unfavorable outcomes 90 days after onset. There was no statistically significant difference in sex, hypertension, diabetes mellitus, heart disease, history of stroke, intraventricular hemorrhage, and lateral ventricle puncture between the favorable outcome group and unfavorable outcome group. However, the unfavorable outcome group had lower GCS scores; higher age, NIHSS scores, APACHE II scores, max-ICH scores, iICH scores, ICH volumes; more days of hospitalization; and a higher proportion of hematoma irregular shape, MIS, and pulmonary infection.

**Table 1:** Patient characteristics of development cohort.

	**mRS ≤ 2 (*n* = 80)**	**mRS > 2 (*n* = 108)**	***P***
Age, median (IQR)	52.0 (46.3–58.0)	55.0 (49.0–65.8)	0.002
Sex, male, *n* (%)	62 (77.5)	77 (71.3)	0.338
Hypertension, *n* (%)	57 (71.3)	85 (78.7)	0.240
Diabetes mellitus, *n* (%)	12 (15.0)	16 (14.8)	0.972
Heart disease, *n* (%)	4 (5.0)	6 (5.6)	>0.999
History of stroke, *n* (%)	10 (12.5)	15 (13.9)	0.782
Days of hospitalization, median (IQR)	10 (7–14)	14 (10–24)	< 0.001
NIHSS, median (IQR)	4 (2–9)	12 (9–16)	< 0.001
APACHE II, median (IQR)	8 (6–11)	12 (8–16)	< 0.001
GCS, median (IQR)	15 (13–15)	11 (9–14)	< 0.001
max-ICH, median (IQR)	1 (0–2)	3 (2–4)	< 0.001
iICH, median (IQR)	3 (1–3)	4 (3–4)	< 0.001
ICH volume, mL, median (IQR)	10.0 (5.5–24.4)	23.9 (15.3–34.6)	< 0.001
IVH, *n* (%)	22 (27.5)	41 (38.0)	0.133
Hematoma irregular shape, *n* (%)	29 (36.3)	70 (64.8)	< 0.001
MIS, *n* (%)	15 (18.8)	52 (48.1)	< 0.001
Lateral ventricle puncture, *n* (%)	6 (7.5)	16 (14.8)	0.123
Pulmonary infection, *n* (%)	24 (30.0)	71 (65.7)	< 0.001
Basal ganglia hemorrhage, *n* (%)	158 (84.0)		
Thalamic hemorrhage, *n* (%)	30 (16.0)		

mRS, modified Rankin Scale; NIHSS, National Institutes of Health Stroke Scale; APACHE II, Acute Physiology and Chronic Health Evaluation II; GCS, Glasgow Coma Scale; max-ICH, maximally treated ICH score; IVH, intraventricular hemorrhage; MIS, minimally invasive surgery.

### 3.3 Multivariable analysis of development cohort

The multivariable logistic regression model was performed to identify predictors of unfavorable outcomes for ICH patients in the development cohort. Variables that showed statistical significance in univariable analysis were considered potential confounding factors (age, days of hospitalization, NIHSS, APACHE II, GCS, ICH volume, hematoma irregular shape, MIS, and pulmonary infection). After adjusting potential confounding factors, iICH showed an independent association with 90-day unfavorable outcomes (Table 2).

**Table 2:** Logistic regression analysis in development cohort.

**Variables**	**Univariable analysis**		**Multivariable analysis** ^*^	

	**Odds ratio (95% CI)**	***P***	**Odds ratio (95% CI)**	***P***
max-ICH	2.955 (2.088–4.181)	< 0.001		0.748
iICH	2.360 (1.752–3.178)	< 0.001	1.958 (1.341–2.858)	0.001

^*^Adjusted for age, days of hospitalization, NIHSS, APACHE II, GCS, ICH volume, hematoma irregular shape, MIS, and pulmonary infection.

### 3.4 Receiver operating characteristic analysis of development cohort

**Figure 3:**
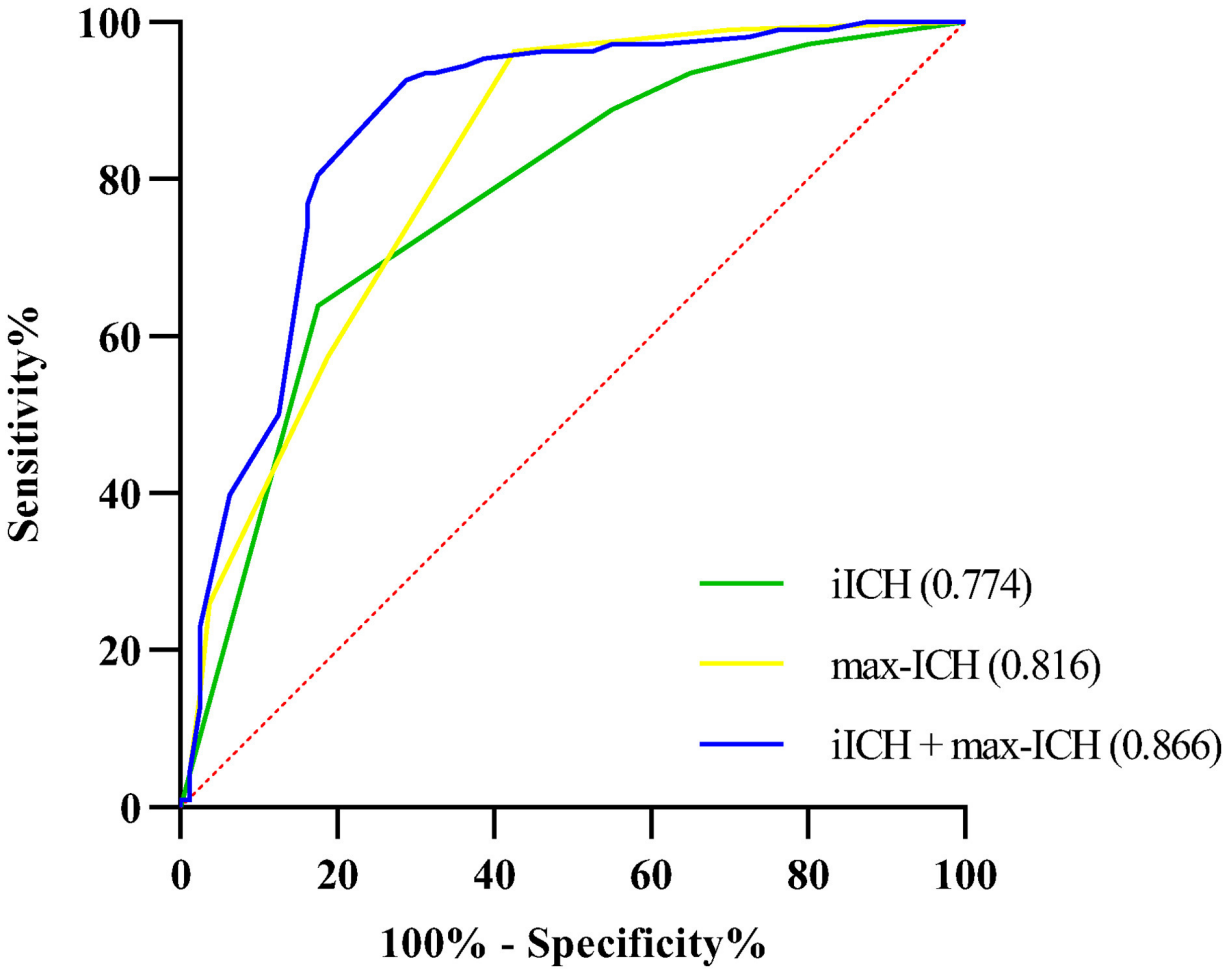
Receiver operating characteristic curves of iICH and max-ICH.

The ROC curve analysis was employed to test the discriminative ability of the above predictors for unfavorable outcomes in ICH patients. Results are shown in Table 3 and Figure 3. The AUCs for max-ICH and iICH are 0.816 (95% CI: 0.753–0.880, *p* < 0.001) and 0.774 (95% CI: 0.705–0.842, *p* < 0.001), respectively. The AUC of max-ICH combined with iICH was 0.866 (95% CI: 0.810–0.922, *p* < 0.001), which was significantly higher than that of max-ICH (DeLong test, *p* = 0.011) and iICH (DeLong test, *p* = 0.002) alone.

**Table 3:** Comparison of discriminative ability by receiver operating characteristic analysis in development cohort.

**Variables**	**AUC (95% CI)**	***P***	**Cut-off value**	**Sensitivity (%)**	**Specificity (%)**
max-ICH	0.816 (0.753–0.880)	< 0.001	1.5	96.3	57.5
iICH	0.774 (0.705–0.842)	< 0.001	3.5	63.9	82.5
max-ICH + iICH	0.866 (0.810–0.922)	< 0.001			

### 3.5 The characteristics of validation cohort

Supplementary Table 1 shows the baseline characteristics of the patients in the validation cohort. During follow-up, 60 (51.3%) patients had unfavorable outcomes 90 days after onset. There was no statistically significant difference in age, sex, hypertension, diabetes mellitus, heart disease, history of stroke, days of hospitalization, intraventricular hemorrhage, and lateral ventricle puncture between the favorable outcome group and unfavorable outcome group. However, the unfavorable outcome group had lower GCS scores; higher NIHSS scores, APACHE II scores, max-ICH scores, iICH scores, and ICH volume; and a higher proportion of hematoma irregular shape and pulmonary infection.

### 3.6 Multivariable analysis of validation cohort

The multivariable logistic regression model was performed to identify predictors of unfavorable outcomes for ICH patients in the validation cohort. Variables that showed statistical significance in univariable analysis were considered potential confounding factors (NIHSS, APACHE II, GCS, ICH volume, hematoma irregular shape, and pulmonary infection). After adjusting potential confounding factors, iICH showed an independent association with 90-day unfavorable outcomes (Supplementary Table 2). In addition, the *p*-values of the Hosmer-Lemeshow test for max-ICH and iICH were 0.923 and 0.162, respectively, which were all >0.05, indicating a good fit.

### 3.7 Receiver operating characteristic analysis of validation cohort

The ROC curve analysis was employed to test the discriminative ability of the above predictors for unfavorable outcomes in ICH patients. Results are shown in Supplementary Table 3. The AUCs for max-ICH and iICH are 0.761 (95% CI: 0.675–0.847, *p* < 0.001) and 0.746 (95% CI: 0.657–0.835, *p* < 0.001), respectively. Similar to the development cohort, the AUC of max-ICH combined with iICH was 0.824 (95% CI: 0.748–0.900, *p* < 0.001), which was significantly higher than that of max-ICH (DeLong test, *p* = 0.047) and iICH (DeLong test, *p* = 0.005) alone.

### 3.8 Validation in MIS subgroup

Utilizing the same statistical analysis methods used in the validation cohort, the results are displayed in Supplementary Table 4.

Of the 67 patients in the MIS subgroup, 52 (77.6%) patients had unfavorable outcomes 90 days after onset. There were no significant differences in all baseline data except for iICH. The unfavorable outcome group had higher iICH scores. The *p*-Value for hypertension was 0.055, which was exceptionally considered a potential confounder. After adjusting potential confounding factors, iICH did not show an independent association with 90-day unfavorable outcomes.

### 3.9 Validation in conservative treatment subgroup

Utilizing the same statistical analysis methods used in the validation cohort, the results are displayed in Supplementary Tables 5–7.

Of the 238 patients in the conservative treatment subgroup, 116 (48.7%) patients had unfavorable outcomes 90 days after onset. There was no statistically significant difference in sex, hypertension, diabetes mellitus, heart disease, and history of stroke between the favorable outcome group and unfavorable outcome group. However, the unfavorable outcome group had lower GCS scores; higher age, NIHSS scores, APACHE II scores, max-ICH scores, iICH scores, and ICH volume; more days of hospitalization; and a higher proportion of intraventricular hemorrhage, hematoma irregular shape, lateral ventricle puncture, and pulmonary infection (Supplementary Table 5).

After adjusting potential confounding factors (age, days of hospitalization, NIHSS, APACHE II, GCS, ICH volume, intraventricular hemorrhage, hematoma irregular shape, lateral ventricle puncture, and pulmonary infection), iICH showed an independent association with 90-day unfavorable outcomes (Supplementary Table 6). In addition, the *p*-values of the Hosmer-Lemeshow test for max-ICH and iICH were 0.379 and 0.071, respectively, indicating a good fit.

As shown in Supplementary Table 7, the AUCs for max-ICH and iICH are 0.820 (95% CI: 0.767–0.872, *p* < 0.001) and 0.769 (95% CI: 0.709–0.829, *p* < 0.001), respectively. The AUC of max-ICH combined with iICH was 0.878 (95% CI: 0.834–0.922, *p* < 0.001), which was significantly higher than that of max-ICH (DeLong test, *p* < 0.001) and iICH (DeLong test, *p* < 0.001) alone.

## 4 Discussion

The main finding of this study is that the involvement of the posterior limb of the internal capsule is closely associated with the prognosis of patients with basal ganglia and thalamic ICH. Reasonably, the posterior limb of the internal capsule is an integrative pathway involved in motor and sensory input, and cerebral hemorrhage in the basal ganglia and thalamic often involves it due to anatomical proximity. We conjectured the involvement of the posterior limb of the internal capsule is a risk factor for forecasting 90 days' prognosis of basal ganglia and thalamic ICH patients, and graded it according to both degrees of compression and damage ulteriorly to obtain the iICH rating scale. The iICH was the result based on a clinical observational study on the development cohort and the same conclusion was reached in the external validation cohort and conservative treatment subgroup, indicating the external validity.

Previous findings have consistently identified risk factors of ICH patients' functional outcome, including volume of hemorrhage, presence of ventricular hemorrhage, location of hemorrhage, age, GCS score, all components of the ICH score (27, 28), sex, midline shift (29), history of arterial hypertension, and baseline blood glucose (30). Unlike the other previous studies, we have taken full advantage of the imaging information and graded the degree of involvement of the posterior limb of the internal capsule based on it. There have been several prospective observational studies that have validated the involvement of the corticospinal tract (including the location of the internal capsule) by multimodal magnetic resonance imaging (MRI), particularly diffusion tensor imaging (DTI), for ICH patients' prognosis (14, 31–34). However, MRI is costly and unavailable in some hospitals. Furthermore, MRI displayed intracerebral hemorrhage is inferior to CT. In this study, the iICH scale is simple and available. The physician only needs to fill in the initial information from the CT scans to derive the appropriate score at the time of admission.

Some of our results are concordant with the previous study on the prediction of functional prognosis in cerebral hemorrhage (Predicting Functional Recovery Scale), where age and NIHSS could be identified as independent predictors of functional outcome (35). However, the imaging parameters in their model had been discarded owing to the lower importance of imaging compared to age and initial NIHSS. Although the volume of hematoma and the presence of intraventricular hemorrhage were not independent risk factors in our study, we did not discard these classic and long-established factors and reflected them by max-ICH. When combined with the iICH score, the AUC of max-ICH was significantly improved, implying that it is a promising indicator for clinical guidance. A multicenter validation study showed that the max-ICH score was consistently superior to the ICH score in the estimation of 3 and 12 months functional outcomes (20). Cheung et al. found that the predictive value of NIHSS for 3-month outcome was higher than any other indicator of stroke severity (36), and it was widely accepted in clinical studies of acute stroke and was able to assess neurological deficits more specifically than the GCS. Therefore, we chose max-ICH rather than ICH for our study. In previous studies, both prospective and retrospective, the area under the curve of max-ICH for predicting mortality in ICH patients was above 0.8, and the area under the curve for predicting functional prognosis was also around 0.8 (20–22), which is consistent with the results of our study. The first external validation of max-ICH in Chinese stroke patients revealed that all items of the max-ICH score were established risk factors of unfavorable long-term (12-month) functional outcomes (22, 37). In this study, we chose the 90-day outcome, rather than a long-term functional outcome due to a prospective study showing that plenty of patients improved dramatically in the first 3 months from severe disability (38). A prospective observational study showed that the max-ICH score showed a good performance in predicting poor functional outcomes at 3 months in the maximal treatment subgroup, with an AUC of 0.86 (95% CI: 0.82–0.90). However, a poor functional outcome was defined as mRS of 4–6 in their study (21).

In this study, a subset of patients from Nanfang Hospital underwent minimally invasive stereotactic hematoma removal, while patients from the other two hospitals were treated conservatively. We divided all patients into the MIS subgroup and conservative treatment subgroup. In the conservative treatment subgroup, max-ICH and iICH showed good predictive value. However, in the MIS subgroup, iICH was not an independent predictor of poor prognosis. To explain this, we hypothesized that the patients in the MIS subgroup had a more severe disease overall (only 12 out of 67 patients got a favorable outcome), which weakened the discrimination of the prediction scales.

There are several limitations to this study. Firstly, it is a retrospective study in which information was retrieved from patient records, which may introduce information bias. Secondly, Many ICH patients who did not have imaging data or whose time of onset was unclear were excluded, potentially resulting in selection bias. Thirdly, different treatment protocols at different centers may also introduce some degree of bias. Fourthly, we did not assess functional status 90 days after the onset, so the predictive value of the involvement of the posterior limb of the internal capsule for long-term prognosis remains unclear. Besides, follow-up by telephone may introduce misjudgments in the assessment of functional outcomes. Finally, although this study is a multicenter study that included three hospitals, the sample size is still insufficient, especially for the MIS subgroups, which may limit the statistical effect. Therefore, prospective studies involving larger populations are necessary to provide evidence for evidence-based medicine.

## 5 Conclusion

Increased involvement of the posterior limb of the internal capsule is associated with a worse outcome in patients with basal ganglia and thalamic ICH. The iICH score has been externally validated to be excellent in predicting the prognosis of patients with basal ganglia and thalamic ICH and may improve the prognostic prediction of the existing scale.

## Data Availability

The raw data supporting the conclusions of this article will be made available by the authors, without undue reservation.
