# Supplementary material for: Involvement of the posterior limb of the internal capsule independently predicts the prognosis of patients with basal ganglia and thalamic hemorrhage
Source: Front Neurol. 2025 Jan 7;15:1475444. doi: 10.3389/fneur.2024.1475444 (PMC11746021; doi:10.3389/fneur.2024.1475444)
Supplement: Supplementary file 1 [file Data_Sheet_1.docx]

**Supplementary Table 1.** Patient characteristics of validation cohort.

|  | mRS ≤ 2 (n = 57) | mRS > 2 (n = 60) | *P* |
| --- | --- | --- | --- |
| Age, median (IQR) | 56.0 (52.5-65.5) | 59.5 (50.0-77.0) | 0.461 |
| Sex, male, n (%) | 32 (56.1) | 41 (68.3) | 0.174 |
| Hypertension, n (%) | 39 (68.4) | 36 (60.0) | 0.343 |
| Diabetes mellitus, n (%) | 8 (14.0) | 5 (8.3) | 0.327 |
| Heart disease, n (%) | 1 (1.8) | 1 (1.7) | >0.999 |
| History of stroke, n (%) | 5 (8.8) | 4 (6.7) | 0.936 |
| Days of hospitalization, median (IQR) | 19 (14-23) | 22 (11-32) | 0.489 |
| NIHSS, median (IQR) | 4 (2-8) | 10 (6-14) | <0.001 |
| APACHE II, median (IQR) | 7 (5-9) | 9 (7-11) | 0.002 |
| GCS, median (IQR) | 15 (13-15) | 13 (11-15) | 0.002 |
| max-ICH, median (IQR) | 1 (1-2) | 3 (2-4) | <0.001 |
| iICH, median (IQR) | 2 (1-3) | 3 (3-4) | <0.001 |
| ICH volume, mL, median (IQR) | 11.7 (4.5-17.1) | 21.2 (13.1-41.4) | <0.001 |
| IVH, n (%) | 17 (29.8) | 20 (33.3) | 0.683 |
| Hematoma irregular shape, n (%) | 17 (29.8) | 30 (50.0) | 0.026 |
| Lateral ventricle puncture, n (%) | 6 (10.5) | 7 (11.7) | 0.844 |
| Pulmonary infection, n (%) | 9 (15.8) | 28 (46.7) | <0.001 |
| Basal ganglia hemorrhage, n (%) | 83 (70.9) | |  |
| Thalamic hemorrhage, n (%) | 34 (29.1) | |  |

Abbreviations: mRS, modified Rankin Scale; NIHSS, National Institutes of Health Stroke Scale; APACHE II, Acute Physiology and Chronic Health Evaluation II; GCS, Glasgow Coma Scale; max-ICH, maximally treated ICH score; IVH, intraventricular hemorrhage.

**Supplementary Table 2.** Logistic regression analysis in validation cohort.

| Variables | Univariable analysis | | Multivariable analysis* | |
| --- | --- | --- | --- | --- |
|  | Odds ratio (95% CI) | *P* | Odds ratio (95% CI) | *P* |
| max-ICH | 1.999 (1.478-2.704) | <0.001 | 1.600 (1.045-2.450) | 0.031 |
| iICH | 2.239 (1.564-3.204) | <0.001 | 1.899 (1.250-2.885) | 0.003 |

*Adjusted for NIHSS, APACHE II, GCS, ICH volume, hematoma irregular shape, and pulmonary infection.

**Supplementary Table 3.** Comparison of discriminative ability by receiver operating characteristic analysis in validation cohort.

| Variables | AUC (95% CI) | *P* | Cut-off value | Sensitivity (%) | Specificity (%) |
| --- | --- | --- | --- | --- | --- |
| max-ICH | 0.761 (0.675-0.847) | <0.001 | 2.5 | 61.7 | 76.8 |
| iICH | 0.746 (0.657-0.835) | <0.001 | 1.5 | 49.3 | 93.3 |
| max-ICH + iICH | 0.824 (0.748-0.900) | <0.001 |  |  |  |

**Supplementary Table 4.** Patient characteristics of MIS subgroup.

|  | mRS ≤ 2 (n = 15) | mRS > 2 (n = 52) | *P* |
| --- | --- | --- | --- |
| Age, median (IQR) | 51.0 (46.0-55.0) | 52.5 (48.0-59.8) | 0.378 |
| Sex, male, n (%) | 12 (80.0) | 37 (71.2) | 0.726 |
| Hypertension, n (%) | 9 (60.0) | 45 (86.5) | 0.055 |
| Diabetes mellitus, n (%) | 3 (20.0) | 6 (11.5) | 0.677 |
| Heart disease, n (%) | 1 (6.7) | 2 (3.8) | >0.999 |
| History of stroke, n (%) | 3 (20.0) | 5 (9.6) | 0.522 |
| Days of hospitalization, median (IQR) | 15 (11-18) | 15 (10-24) | 0.493 |
| NIHSS, median (IQR) | 11 (8-17) | 13 (10-16) | 0.751 |
| APACHE II, median (IQR) | 7 (5-9) | 9 (7-11) | 0.397 |
| GCS, median (IQR) | 10 (8-12) | 10 (9-12) | 0.475 |
| max-ICH, median (IQR) | 3 (2-3) | 3 (2-3) | 0.845 |
| iICH, median (IQR) | 3 (3-3) | 4 (3-4) | 0.017 |
| ICH volume, mL, median (IQR) | 27.3 (22.8-35.0) | 31.0 (23.5-42.9) | 0.332 |
| IVH, n (%) | 2 (13.3) | 11 (21.2) | 0.761 |
| Hematoma irregular shape, n (%) | 10 (66.7) | 32 (61.5) | 0.718 |
| Lateral ventricle puncture, n (%) | 1 (6.7) | 1 (1.9) | 0.928 |
| Pulmonary infection, n (%) | 11 (73.3) | 40 (76.9) | >0.999 |
| Basal ganglia hemorrhage, n (%) | 66 (98.5) | |  |
| Thalamic hemorrhage, n (%) | 1 (1.5) | |  |

Abbreviations: mRS, modified Rankin Scale; NIHSS, National Institutes of Health Stroke Scale; APACHE II, Acute Physiology and Chronic Health Evaluation II; GCS, Glasgow Coma Scale; max-ICH, maximally treated ICH score; IVH, intraventricular hemorrhage.

**Supplementary Table 5.** Patient characteristics of conservative treatment subgroup.

|  | mRS ≤ 2 (n = 122) | mRS > 2 (n = 116) | *P* |
| --- | --- | --- | --- |
| Age, median (IQR) | 54.0 (49.0-63.0) | 60.0 (51.0-71.0) | 0.002 |
| Sex, male, n (%) | 82 (67.2) | 81 (69.8) | 0.664 |
| Hypertension, n (%) | 87 (71.3) | 76 (65.5) | 0.336 |
| Diabetes mellitus, n (%) | 17 (13.9) | 15 (12.9) | 0.821 |
| Heart disease, n (%) | 4 (3.3) | 5 (4.3) | 0.939 |
| History of stroke, n (%) | 12 (9.8) | 14 (12.1) | 0.581 |
| Days of hospitalization, median (IQR) | 13 (9-19) | 16 (10-28) | 0.007 |
| NIHSS, median (IQR) | 4 (2-7) | 11 (7-15) | <0.001 |
| APACHE II, median (IQR) | 7 (5-9) | 10 (7-15) | <0.001 |
| GCS, median (IQR) | 15 (14-15) | 13 (10-15) | <0.001 |
| max-ICH, median (IQR) | 1 (0-2) | 3 (2-4) | <0.001 |
| iICH, median (IQR) | 2 (1-3) | 4 (3-4) | <0.001 |
| ICH volume, mL, median (IQR) | 9.6 (4.6-17.1) | 19.0 (11.2-31.8) | <0.001 |
| IVH, n (%) | 37 (30.3) | 50 (43.1) | 0.041 |
| Hematoma irregular shape, n (%) | 36 (29.5) | 68 (58.6) | <0.001 |
| Lateral ventricle puncture, n (%) | 11 (9.0) | 22 (19.0) | 0.026 |
| Pulmonary infection, n (%) | 22 (18.0) | 59 (50.9) | <0.001 |
| Basal ganglia hemorrhage, n (%) | 175 (73.5) | |  |
| Thalamic hemorrhage, n (%) | 63 (26.5) | |  |

Abbreviations: mRS, modified Rankin Scale; NIHSS, National Institutes of Health Stroke Scale; APACHE II, Acute Physiology and Chronic Health Evaluation II; GCS, Glasgow Coma Scale; max-ICH, maximally treated ICH score; IVH, intraventricular hemorrhage.

**Supplementary Table 6.** Logistic regression analysis in conservative treatment subgroup.

| Variables | Univariable analysis | | Multivariable analysis* | |
| --- | --- | --- | --- | --- |
|  | Odds ratio (95% CI) | *P* | Odds ratio (95% CI) | *P* |
| max-ICH | 2.607 (2.013-3.377) | <0.001 |  | 0.180 |
| iICH | 2.311 (1.795-2.976) | <0.001 | 2.098 (1.516-2.904) | <0.001 |

*Adjusted for age, days of hospitalization, NIHSS, APACHE II, GCS, ICH volume, intraventricular hemorrhage, hematoma irregular shape, lateral ventricle puncture, and pulmonary infection.

**Supplementary Table 7.** Comparison of discriminative ability by receiver operating characteristic analysis in conservative treatment subgroup.

| Variables | AUC (95% CI) | *P* | Cut-off value | Sensitivity (%) | Specificity (%) |
| --- | --- | --- | --- | --- | --- |
| max-ICH | 0.820 (0.767-0.872) | <0.001 | 1.5 | 87.9 | 62.3 |
| iICH | 0.769 (0.709-0.829) | <0.001 | 3.5 | 86.1 | 55.2 |
| max-ICH + iICH | 0.878 (0.834-0.922) | <0.001 |  |  |  |
